# Ovarian Response, Pregnancy Outcomes, and Complications Between Salpingectomy and Proximal Tubal Occlusion in Hydrosalpinx Patients Before *in vitro* Fertilization: A Meta-Analysis

**DOI:** 10.3389/fsurg.2022.830612

**Published:** 2022-04-29

**Authors:** Hua Ou, Jie Sun, Lin Lin, Xiao Ma

**Affiliations:** ^1^Medical Examination Center, China-Japan Friendship Hospital, Beijing, China; ^2^Department of Gynecology and Obstetrics, China-Japan Friendship Hospital, Beijing, China; ^3^Department of Gynecology and Obstetrics, The Maternal and Child Health Hospital of Guangxi Zhuang Autonomous Region, Nanning, China

**Keywords:** hydrosalpinx before *in vitro* fertilization, salpingectomy and proximal tubal occlusion, ovarian response, pregnancy outcomes, complication

## Abstract

**Objectives:**

Contradictory findings exist in studies comparing salpingectomy and proximal tubal occlusion (PTO) in treating hydrosalpinx patients before *in vitro* fertilization (IVF). Therefore, this meta-analysis aimed to comprehensively compare ovarian response, pregnancy outcomes, and complications between salpingectomy and PTO in treating these patients.

**Methods:**

Embase, PubMed, and Web of Science were searched to identify relevant articles published from 1980 to August 31, 2020. Eight studies that involve 716 hydrosalpinx patients before IVF were included, among whom 408 patients received salpingectomy and 308 patients received PTO. The data were pooled; the standardized mean difference (SMD) or odds ratio (OR) was calculated.

**Results:**

Proximal tubal occlusion-treated patients had higher fertilization rate (SMD = 0.35, 95% CI: 0.11–0.59), while similar days of controlled ovarian hyperstimulation (COH) (SMD: 0.15, 95% CI: −0.36–0.67) and number of retrieved oocytes (SMD = −0.22, 95% CI: −0.54–0.10) compared with salpingectomy-treated patients. Furthermore, no difference of implantation rate (OR = 1.17, 95% CI: 0.62–2.20), clinical pregnancy rate (OR = 0.82, 95% CI: 0.59–1.15), ongoing pregnancy rate (OR = 0.64, 95% CI: 0.36–1.13), or live birth rate (OR = 0.67, 95% CI: 0.16–2.72) was shown between salpingectomy-treated patients and PTO-treated patients. Additionally, ectopic pregnancy rate (OR = 1.13, 95% CI: 0.21–5.92) and miscarriage rate (OR = 0.88, 95% CI: 0.31–2.48) were similar between salpingectomy-treated patients and PTO-treated patients.

**Conclusion:**

Proximal tubal occlusion exhibits a higher fertilization rate but no obvious benefits on days of COH, number of retrieved oocytes, pregnancy outcomes, and complications over salpingectomy in hydrosalpinx patients before IVF.

## Introduction

Hydrosalpinx, a common condition of female infertility, is marked by a distally blocked, dilated, fluid-filled fallopian tube, accounting for approximately 30% of patients undergoing *in vitro* fertilization (IVF) (1, 2). Hydrosalpinx deteriorates the IVF outcomes, such as lower implantation rates, pregnancy, increased risks of early pregnancy loss, and ectopic pregnancies, due to altered embryotoxic properties, impaired endometrial receptivity, or hydrosalpingeal fluid mechanically flushing the embryo (1, 3). Salpingectomy is the most widely used intervention for removing chronically infected fallopian tubes in managing hydrosalpinx before IVF (1). While the interfered ovarian blood flow following salpingectomy is reported to increase the risk of damage to major organs or vessels, increase the risk of interstitial pregnancy, and decline ovarian response to gonadotropin stimulation in salpingectomy-treated hydrosalpinx patients before IVF (1). Therefore, exploring a less invasive alternative management approach is essential for improving ovarian response, IVF outcomes and reducing complications in the management of hydrosalpinx patients prior to IVF.

As a less invasive approach, proximal tubal occlusion (PTO) is easier and quicker to perform, eliminate the hydrosalpingeal fluid's retrograde flow into the endometrial cavity, and preserve the ovarian blood supply in hydrosalpinx patients prior to IVF (3). Earlier literature compares salpingectomy and PTO's effect on the ovarian response, IVF outcomes, and/or complications in treating hydrosalpinx patients prior to IVF (4–11). For instance, Malhotra et al. elucidate no difference between salpingectomy and PTO regarding fertilization, clinical pregnancy, ongoing pregnancy, or miscarriage rate in hydrosalpinx patients prior to IVF (10). Whereas, Vignarajan et al. illuminate that fertilization rate is increased in PTO-treated hydrosalpinx patients compared to salpingectomy-treated hydrosalpinx patients prior to IVF (8). To address the controversial findings, a meta-analysis of published data relating to comparing ovarian response, IVF outcomes, and complications between salpingectomy and PTO in treating hydrosalpinx patients prior to IVF is necessary. Although there is one previous relevant meta-analysis, i.e., 357 hydrosalpinx patients prior to IVF from four articles in 2015, the numbers of included patients and articles are relatively small; furthermore, the complications are not compared (12).

Therefore, we conducted this meta-analysis that include data from eight studies with 716 hydrosalpinx patients prior to IVF to compare ovarian response, IVF outcomes, and complications between salpingectomy and PTO.

## Methods

### Data Sources and Search Strategy

A literature search in Embase, PubMed, and Web of Science from 1980 to August 31, 2020 was performed to identify the published cohorts (retrospective or prospective) or randomized controlled trials (RCTs) comparing efficacy or safety between salpingectomy and PTO for hydrosalpinx patients prior to IVF. The following keywords were used in combination for searching: “*hydrosalpinx,” “hydrosalpinges,” “hydrosalpingeal fluid,” “hydrops tubae,” “hydrops tubae profluens,” “tubal occlusion,” “salpingectomy,”* and “*tubectomy.”* This meta-analysis was carried out by the Preferred Reporting Items for Systematic Reviews and Meta-Analyses statement and the Meta-analysis Of Observational Studies in Epidemiology guidelines (13).

### Selection of Studies

The retrieved articles were first filtered by title reviewing and then screened by abstract or full-text reviewing. The studies met all the following criteria were eligible to be included in the analysis: (1) patients had a diagnosis of hydrosalpinx; (2) published cohorts (retrospective or prospective) or RCTs comparing efficacy or safety between salpingectomy and PTO for hydrosalpinx patients prior to IVF; (3) the study included at least one of the following outcomes: days of controlled ovarian hyperstimulation (COH), the number of retrieved oocytes, fertilization rate, clinical pregnancy rate, ongoing pregnancy rate, implantation rate, live birth rate, miscarriage rate, or ectopic pregnancy rate; (4) English articles or abstract; (5) outcome data were available from full text or abstract. Reviews and case reports, duplicated data, experimental studies, non-comparative studies, or the studies without available data were excluded. In addition, we examined the reference lists of the retrieved studies and carefully reviewed articles from the bibliographic database.

### Data Extraction

Data extraction was performed independently by two reviewers. The data were filtered and screened into a standard electronic form. In case of disagreement, a consensus was reached after a discussion between the two reviewers. When getting an agreement was difficult, the principal investigator made the final decision on the study's eligibility and the data extraction. The following data were extracted from each of the eligible studies: the first author's name, the year of publication, methodology (study design), number of patients, surgical intervention, and the following outcomes: days of COH, retrieved oocytes, fertilization rate, clinical pregnancy rate, ongoing pregnancy rate, implantation rate, live birth rate, miscarriage rate, and ectopic pregnancy rate.

### Statistical Analysis

Meta-analysis was performed using a meta package in R 3.6.3 software (Comprehensive R Archive Network, USA). Among all comparisons, the patients treated with salpingectomy were considered the control group, while the patients treated with PTO were considered the experimental group. The continuous variables for meta-analysis were expressed as a standardized mean difference (SMD) with 95% CI. Categorized variables were expressed as odds ratios (ORs) with 95% CI.

Heterogeneity between studies was assessed by the *I*^2^-test, and an *I*^2^ > 50% indicated significant heterogeneity between studies. When heterogeneity was significant (*I*^2^ > 50%), the random-effect model was used for meta-analysis; when there was no significant heterogeneity (*I*^2^ ≤ 50%), the fixed-effect meta-analysis was used. The potential publication bias was analyzed by funnel plot and determined by the Egger regression test and Begg and Mazumdar test for meta-analysis with more than four Studies.

## Results

### Study Selection Procedure

**Figure 1:**
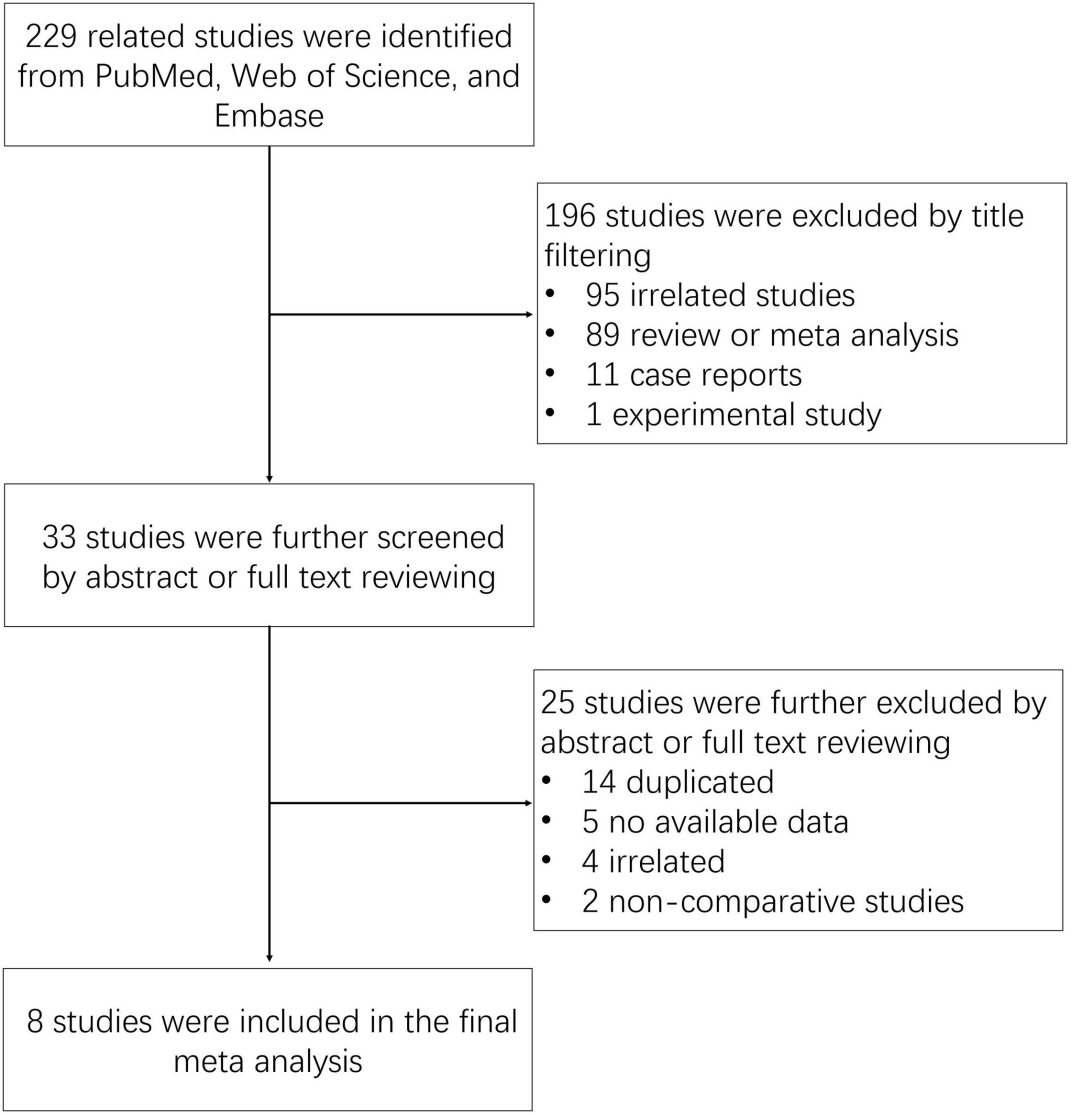
Flowchart of study selection.

The search strategy identified 229 related studies from PubMed, Web of Science, and Embase (Figure 1). After reviewing the title, a total of 196 studies (95 unrelated studies, 89 reviews or meta-analyses, 11 case reports, and one experimental study) were excluded. The remaining 33 studies were filtered by abstract or full-text review. Among these studies, 25 studies (14 duplicated studies, five without available data, four unrelated studies, and two non-comparative studies) were excluded. In the final meta-analysis, eight studies were included.

### Characteristics of Included Studies

The main characteristics of the eight included studies are summarized in Table 1. All studies were published from 2001 to 2020, and there were three RCT studies from India, one RCT from the Netherlands, one RCT study from Greece, two cohort studies from the USA, and one cohort study from China. Additionally, a total of 716 hydrosalpinx patients prior to IVF were included, among whom 408 patients received salpingectomy prior to IVF whereas 308 patients received PTO prior to IVF.

**Table 1:** Characteristics of studies included in the meta-analysis.

**References**	**Year**	**Country**	**Study design**	**No. of patients**			**Clinical pregnancy rate (%)**		**Ectopic pregnancy rate (%)**		**Miscarriage rate (%)**	
				**Total**	**Salpingectomy**	**PTO**	**Salpingectomy**	**PTO**	**Salpingectomy**	**PTO**	**Salpingectomy**	**PTO**
Yang et al. (9)	2020	China	Cohort	155	113	42	46.0	40.5	3.5	2.4	2.7	2.4
Vignarajan et al. (8)	2019	India	RCT	165	82	83	25.6	33.7	N/A	N/A	4.8	3.6
Dreyer et al. (7)	2016	Netherlands	RCT	85	43	42	58.1	31.0	0.0	0.0	2.6	1.7
Malhotra and Vignarajan (10)	2015	India	RCT	75	38	37	18.4	24.3	N/A	N/A	0.0	2.7
Malhotra et al. (11)	2014	India	RCT	72	35	37	N/A	N/A	N/A	N/A	N/A	N/A
Kontoravdis et al. (6)	2006	Greece	RCT	92	47	45	55.3	44.4	0.0	2.2	6.4	4.4
Sagoskin et al. (5)	2003	USA	Cohort	25	18	7	88.9	85.7	N/A	N/A	N/A	N/A
Surrey and Schoolcraft (4)	2001	USA	Cohort	47	32	15	57.1	46.7	N/A	N/A	N/A	N/A

*PTO, proximal tubal occlusion; USA, United States of America; RCT, randomized controlled trial; N/A, not available*.

### Days of COH, Number of Retrieved Oocytes, and Fertilization Rate

**Figure 2:**
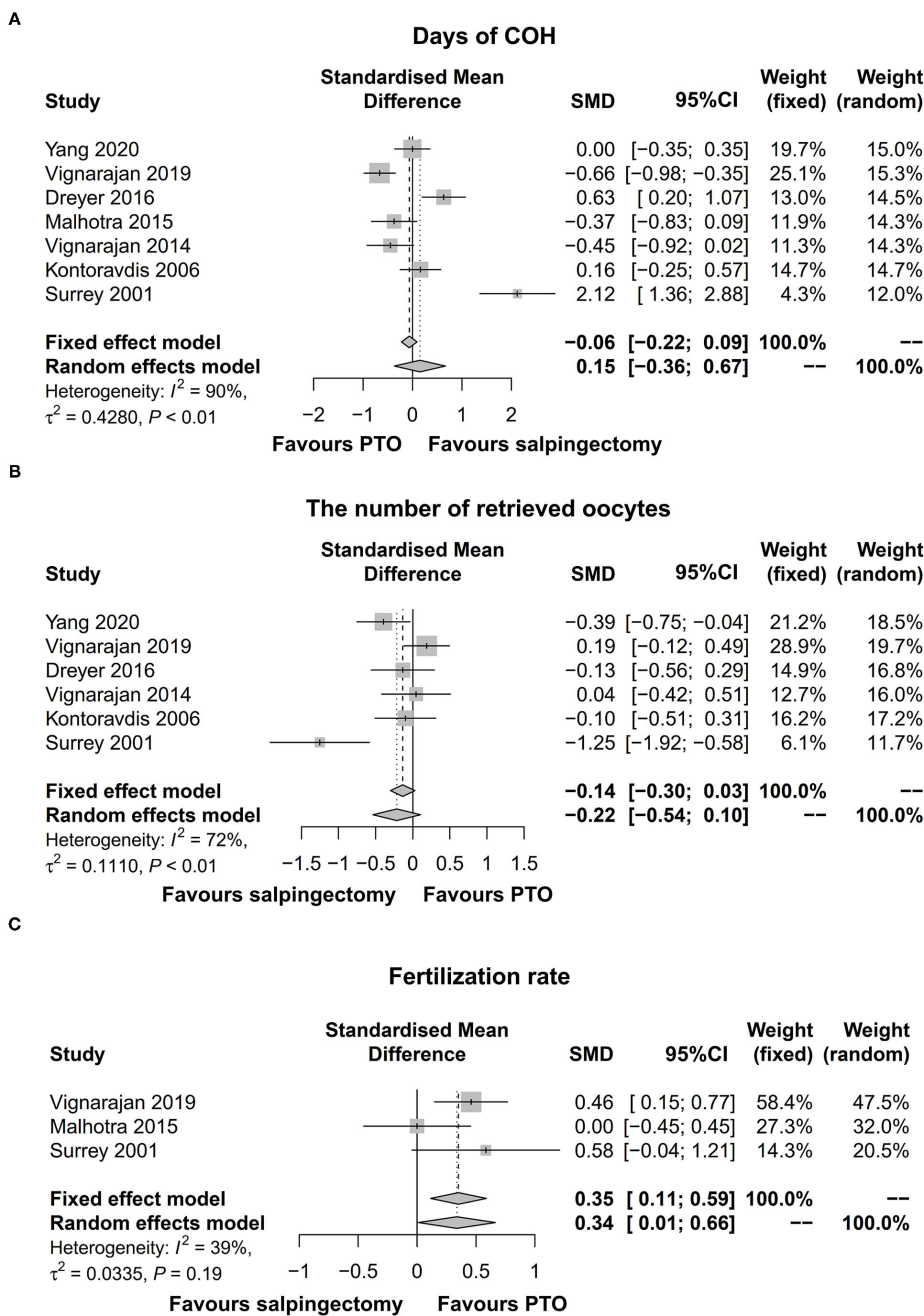
Forest plot comparing days of COH **(A)**, the number of retrieved oocytes **(B)**, and fertilization rate **(C)** between salpingectomy-treated hydrosalpinx patients and PTO-treated hydrosalpinx patients prior to IVF. COH, controlled ovarian hyperstimulation; PTO-treated, proximal tubal occlusion-treated; IVF, *in vitro* fertilization.

Totally, seven studies provided data on days of COH; 6 studies displayed data on the number of retrieved oocytes; and three studies presented data on fertilization rate. The pooled analyses showed that days of COH [SMD (95% CI) = 0.15 (−0.36; 0.67)] (Figure 2A) and number of retrieved oocytes [SMD (95% CI) = −0.22 [−0.54; 0.10)] (Figure 2B) did not differ between salpingectomy group and PTO group prior to IVF with significant heterogeneity among studies (days of COH: *I*^2^ = 90%; number of retrieved oocytes: *I*^2^ = 72%), while fertilization rate [SMD (95% CI) = 0.35 (0.11; 0.59)] (Figure 2C) was obviously increased in PTO group compared to salpingectomy group prior to IVF without significant heterogeneity among studies (*I*^2^ = 39%).

### Implantation, Clinical Pregnancy, and Ongoing Pregnancy Rates

**Figure 3:**
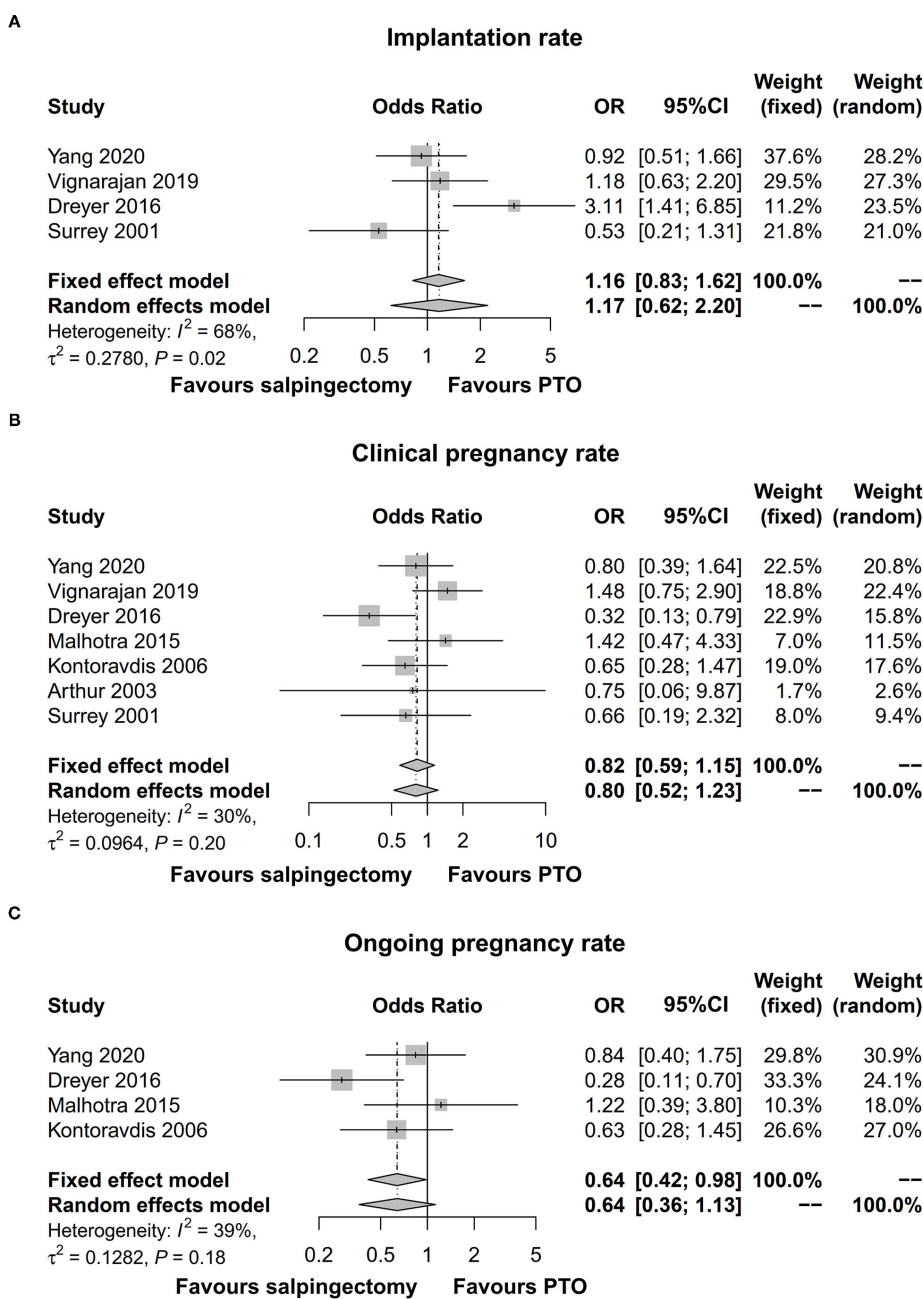
Forest plot comparing implantation rate **(A)**, clinical pregnancy rate **(B)**, and ongoing pregnancy rate **(C)** between salpingectomy-treated hydrosalpinx patients and PTO-treated hydrosalpinx patients prior to IVF. PTO-treated, proximal tubal occlusion-treated; IVF, *in vitro* fertilization.

Four studies presented data on implantation rate and ongoing pregnancy rate and seven studies exhibited data on clinical pregnancy rate. The pooled analyses revealed that no difference of implantation rate [OR (95% CI) = 1.17 (0.62; 2.20)] (*I*^2^ = 68%, significant heterogeneity among studies) (Figure 3A), clinical pregnancy rate [OR (95% CI) = 0.82 (0.59; 1.15)] (*I*^2^ = 30%, no significant heterogeneity among studies) (Figure 3B), or ongoing pregnancy rate [OR (95% CI) = 0.64 (0.36; 1.13)] (*I*^2^ = 39%, no significant heterogeneity among studies) (Figure 3C) was shown between salpingectomy group and PTO group prior to IVF.

### Live Birth Rate

**Figure 4:**
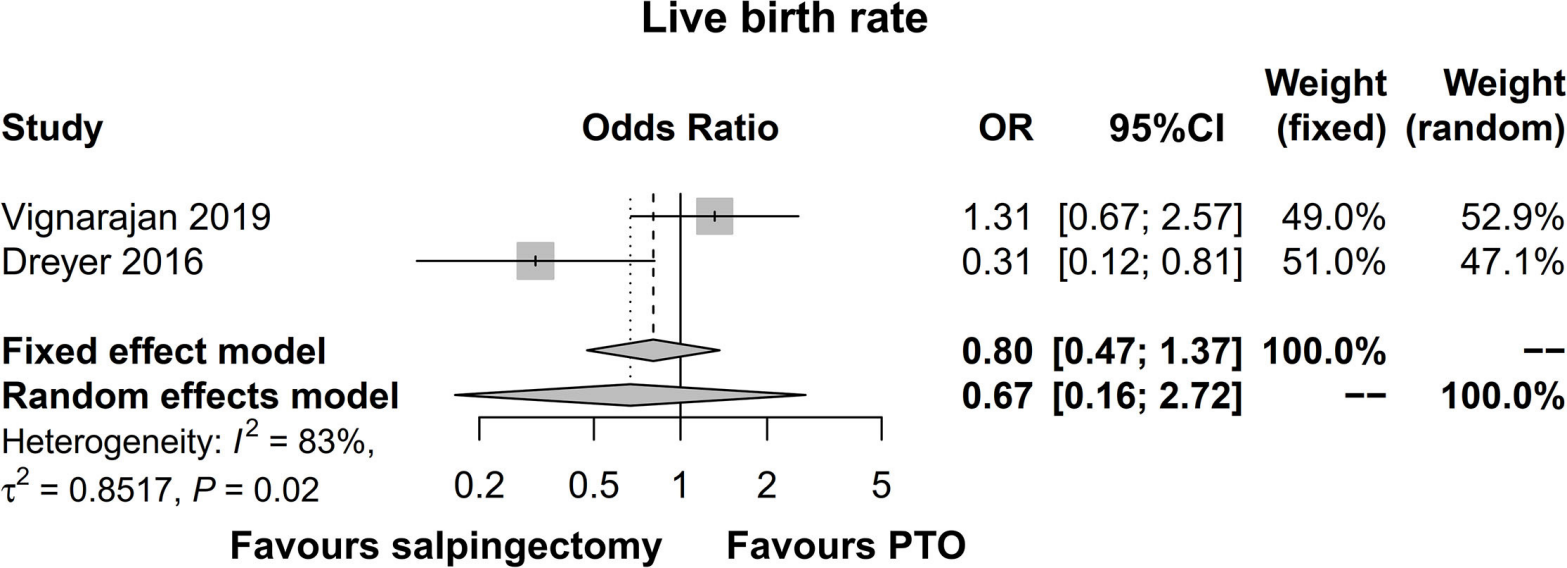
Forest plot comparing live birth rate between salpingectomy-treated hydrosalpinx patients and PTO-treated hydrosalpinx patients prior to IVF. PTO-treated, proximal tubal occlusion-treated; IVF, *in vitro* fertilization.

Two studies presented data on live birth rate, which were included in the pooled analysis. The pooled analysis disclosed that the live birth rate was not different in the salpingectomy group compared with the PTO group prior to IVF [OR (95% CI) = 0.67 (0.16; 2.72)] (Figure 4). Meanwhile, there was significant heterogeneity between the two studies (*I*^2^ = 83%).

### Ectopic Pregnancy Rate and Miscarriage Rate

**Figure 5:**
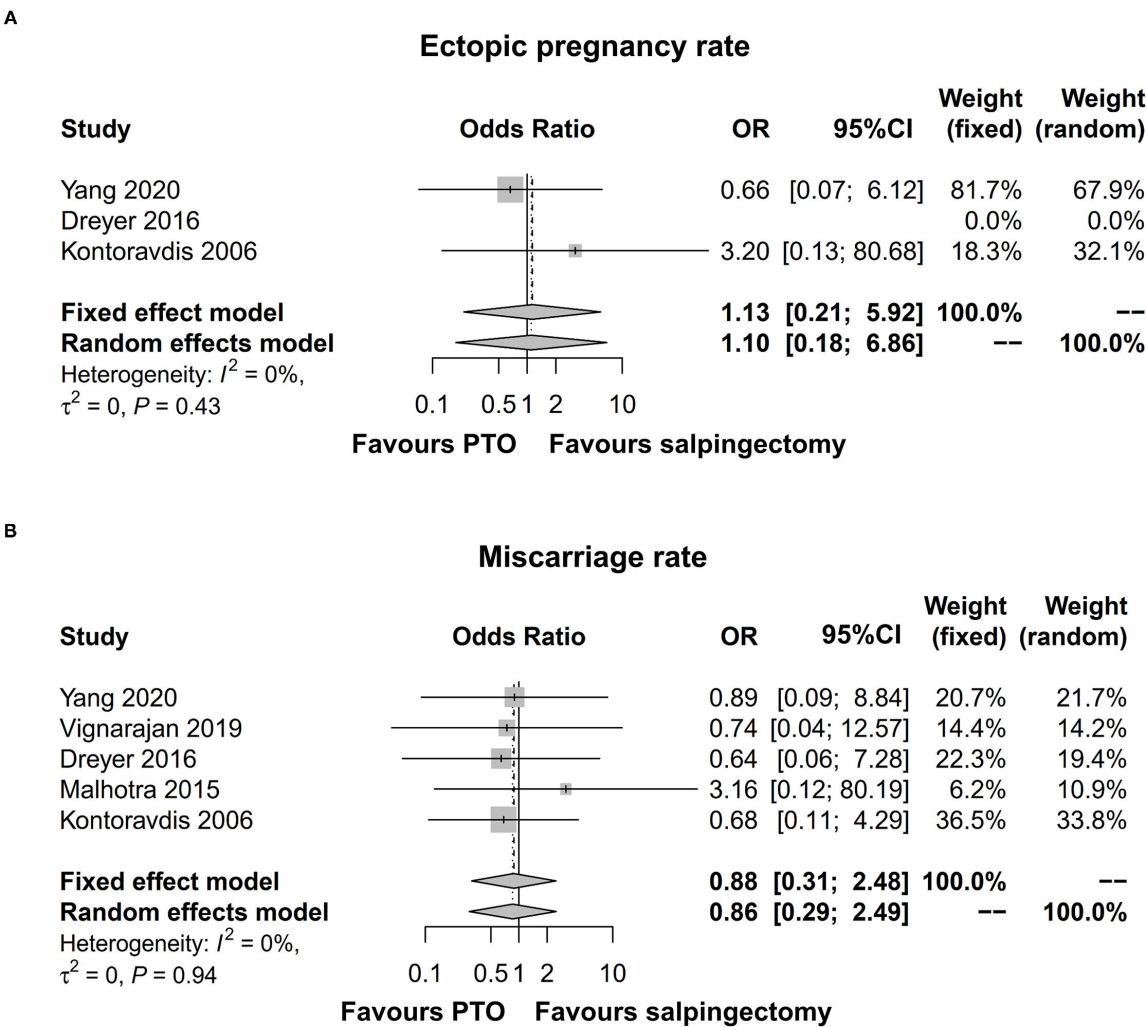
Forest plot comparing ectopic pregnancy rate **(A)** and miscarriage rate **(B)** between salpingectomy-treated hydrosalpinx patients and PTO-treated hydrosalpinx patients prior to IVF. PTO-treated, proximal tubal occlusion-treated; IVF, *in vitro* fertilization.

Three studies exhibited data on ectopic pregnancy rate and five studies displayed data on miscarriage rate. The pooled analysis illuminated that ectopic pregnancy rate [OR (95% CI) = 1.13 (0.21; 5.92)] (Figure 5A) or miscarriage rate [OR (95% CI) = 0.88 (0.31; 2.48)] (Figure 5B) was of no differences in salpingectomy group compared to PTO group prior to IVF. Meanwhile, no significant heterogeneity was noted among studies (ectopic pregnancy rate: *I*^2^ = 0%; miscarriage rate: *I*^2^ = 0%).

### Publication Bias

Initially, potential publication bias was analyzed by funnel plot, which displayed that the shape of funnel plot was not obviously asymmetric in days of COH, the number of retrieved oocytes, fertilization rate, implantation rate, clinical pregnancy rate, ongoing pregnancy, liver birth rate, ectopic pregnancy rate, or miscarriage rate (Supplementary Figures 1A–I). Then, the Egger test and Begg and Mazumdar test were used to determine potential publication bias of meta-analysis with more than four studies, which revealed that no publication bias was observed in days of COH, the number of retrieved oocytes, clinical pregnancy rate, or miscarriage rate across the included studies (Egger test: all *p* ≥ 0.05; Begg and Mazumdar test: all *p* ≥ 0.05; Supplementary Figures 1A,B,E,I).

## Discussion

The conclusions are contradictory in prior studies comparing ovarian response, IVF treatment outcomes, and complications between salpingectomy and PTO in hydrosalpinx patients prior to IVF. In the current meta-analysis, a total of 8 studies that involve 716 hydrosalpinx patients prior to IVF were included, of whom 408 patients received salpingectomy and 308 patients received PTO. The pooled results of ovarian response and IVF treatment outcomes showed that fertilization rate was higher, while days of COH, number of retrieved oocytes, implantation rate, clinical pregnancy rate, ongoing pregnancy rate, and live birth rate were similar in PTO-treated hydrosalpinx patients compared with salpingectomy-treated hydrosalpinx patients prior to IVF. The findings could be explained by that (i) salpingectomy removed the chronically infected fallopian tube to eliminate hydrosalpinx fluid, while it might potentially interrupt ovarian blood supply, compromised ovarian blood flow, declined ovarian function reserve, and declining endometrial receptivity; PTO (blocked the junction of fallopian tube and uterus) eliminated hydrosalpinx fluid retrograde flow in the uterine cavity, preserved ovarian blood supply, and did not impact ovarian function, meanwhile, the better ovarian function potentially resulted in more active retrieved oocytes when fertilized with sperm *in vitro* and a higher chance of fertilization; taken together, PTO displayed higher fertilization rate than salpingectomy in treating hydrosalpinx patients prior to IVF (1, 3, 14, 15). (ii) Both salpingectomy (removal of the infected fallopian tube) and PTO (blockage at the junction of fallopian tube and uterus) eliminated the toxic hydrosalpinx fluid retrograde flow in the uterine cavity, which probably improved the access to the ovary, enhanced the optimization of oocyte-retrieved conditions, increased endometrial receptivity, and facilitated fertilization and pregnancy, therefore, salpingectomy and PTO exhibited no difference on days of COH, number of retrieved oocytes, implantation rate, clinical pregnancy rate, and live birth rate in treating hydrosalpinx patients prior to IVF (1, 3, 16, 17).

After salpingectomy, dense adhesions, interstitial pregnancy, and injury to the urinary tract are commonly reported complications in hydrosalpinx patients prior to IVF (3). Although PTO is a less invasive approach, it may be linked with pelvic pain, presence of diseased tube, and risk of tubal surgery in PTO-treated hydrosalpinx patients prior to IVF (1). In the current meta-analysis, the pooled results of complications exhibited that ectopic pregnancy rate and miscarriage rate were similar between salpingectomy-treated hydrosalpinx patients and PTO-treated hydrosalpinx patients prior to IVF. The following were the possible explanations: salpingectomy might potentially induce injury to surrounding structures, which reduced endometrial receptivity; meanwhile, during the PTO treatment, the presence of micro-inserts (e.g., coils) inside the endometrial cavity might be linked with lower endometrial receptivity, perforation, and intraperitoneal migration, which resulted in the development of an intrauterine fluid collection and decreased endometrial receptivity; taken together, both salpingectomy and PTO could potentially lead to ectopic pregnancy and miscarriage, hence, ectopic pregnancy rate or miscarriage rate did not differ in salpingectomy-treated hydrosalpinx patients compared with PTO-treated hydrosalpinx patients prior to IVF (4, 16–19).

It could be mentioned that woman's age and ovarian reserve markers are considered as tools to optimize the recombinant follicle-stimulating hormone (rFSH) starting dose in IVF procedure, while the evidence is not adequate. An interesting study constructs a nomogram based on a woman's age and ovarian reserve markers, which provide a more tailored FSH starting dose and improved cost-effectiveness; while not so good in polycystic ovary syndrome women especially the ones with high anti-Müllerian hormone (20). While another study observes that ovarian reserve markers fail to predict the hypo-response in normovulatory infertile women (21). In addition, it is also discovered that larger amounts of gonadotropins are needed in obese women compared to normal weighting women in order to realize a similar IVF success rate (22).

Nevertheless, the findings of the current meta-analysis should be interpreted in the context of some limitations. First, the variations in publication dates (ranging from 2001 to 2020, with significant changes in IVF treatment and embryological protocols), study design, inclusion criteria/exclusion criteria of hydrosalpinx patients, surgical techniques, and operation skills of surgeons of different studies might result in confounding biases of the results. Second, only 2 studies displayed live birth rate, and 3 studies presented with fertilization data/ectopic rate data, the limited data might decrease the statistical power of the meta-analysis, therefore, further validating studies should be warranted. Lastly, the limited number of RCT studies might lead to the existence of confounding factors, hence, additional RCTs were needed in the future for validating the findings of the current meta-analysis.

To sum up, PTO is superior to salpingectomy regarding fertilization rate while displays no obvious benefits on days of COH, number of retrieved oocytes, IVF outcomes, and complications in treating hydrosalpinx patients prior to IVF. These findings might offer guidance for physicians in proper treatment selection in the management of hydrosalpinx prior to IVF.

## Data Availability Statement

The original contributions presented in the study are included in the article/Supplementary Material, further inquiries can be directed to the corresponding author.

## Author Contributions

XM conceived and designed the study and supervised the project. HO and XM performed the experiments. JS and LL analyzed, interpreted data, and drafted the manuscript. All authors participated in the writing, revision of the manuscript, read, and approved the final manuscript.

## Conflict of Interest

The authors declare that the research was conducted in the absence of any commercial or financial relationships that could be construed as a potential conflict of interest.

## Publisher's Note

All claims expressed in this article are solely those of the authors and do not necessarily represent those of their affiliated organizations, or those of the publisher, the editors and the reviewers. Any product that may be evaluated in this article, or claim that may be made by its manufacturer, is not guaranteed or endorsed by the publisher.
