# Supplementary material for: Ovarian Response, Pregnancy Outcomes, and Complications Between Salpingectomy and Proximal Tubal Occlusion in Hydrosalpinx Patients Before in vitro Fertilization: A Meta-Analysis
Source: Front Surg. 2022 Apr 29;9:830612. doi: 10.3389/fsurg.2022.830612 (PMC9099031; doi:10.3389/fsurg.2022.830612)
Supplement: Supplementary file 1 [file Data_Sheet_1.PDF]

Supplementary Material

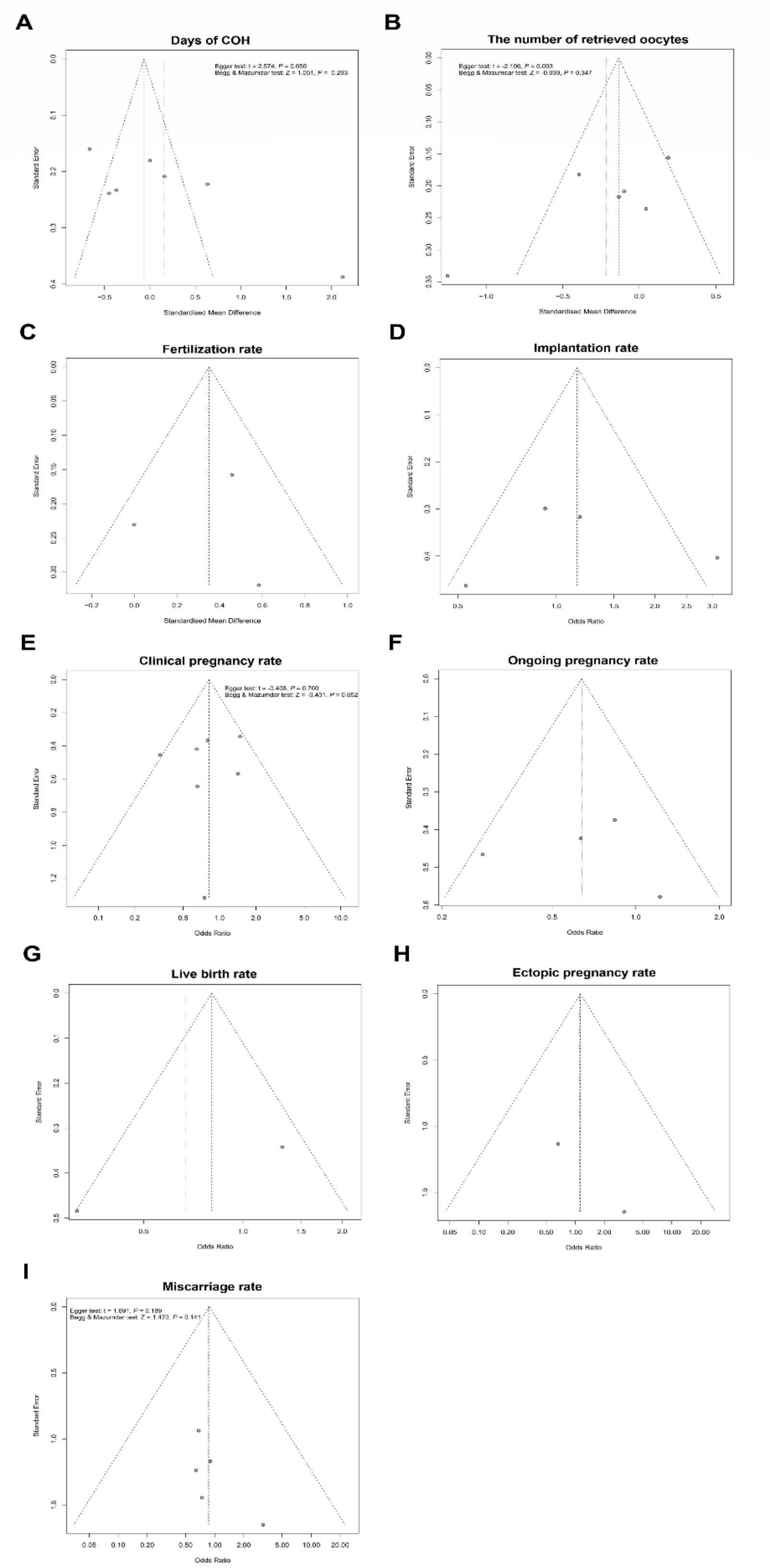

Supplementary figure 1. Funnel plot, Egger regression test and Begg & Mazumdar test for the publication bias test of included studies regarding days of COH (**A**), number of retrieved oocytes (**B**), fertilization rate (**C**), implantation rate (**D**), clinical pregnancy rate (**E**), ongoing pregnancy (**F**), liver birth rate (**G**), ectopic pregnancy rate (**H**) and miscarriage rate (**I**). COH, controlled ovarian hyperstimulation in hydrosalpinx patients prior to IVF. IVF, *in vitro* fertilization.
